# Supplementary material for: Voltage-clamp fluorometry analysis of structural rearrangements of ATP-gated channel P2X2 upon hyperpolarization
Source: eLife. 2021 May 19;10:e65822. doi: 10.7554/eLife.65822 (PMC8184218; doi:10.7554/eLife.65822)

ANOVAOneWay (23/12/2020 15:20:02)

Input Data

	Data	Range
Control	[Book1]Sheet1!A"Control"	[1*:8*]
30 nM SIK	[Book1]Sheet1!B"30 nM SIK"	[1*:6*]
300 nM SIK	[Book1]Sheet1!C"300 nM SIK"	[1*:6*]

Descriptive Statistics

	N Analysis	N Missing	Mean	Standard Deviation	SE of Mean
Control	8	0	3.1975	2.36339	0.83559
30 nM SIK	6	0	6.39833	4.6318	1.89092
300 nM SIK	6	0	10.60167	6.04239	2.4668

One Way ANOVA

Overall ANOVA

	DF	Sum of Squares	Mean Square	F Value	Prob>F
Model	2	187.96326	93.98163	4.85738	0.02145
Error	17	328.91992	19.34823		
Total	19	516.88318			

Null Hypothesis: The means of all levels are equal.
Alternative Hypothesis: The means of one or more levels are different.
At the 0.05 level, the population means are significantly different.

Fit Statistics

	R-Square	Coeff Var	Root MSE	Data Mean
	0.36365	0.68955	4.39866	6.379

Means Comparisons

Tukey Test

	MeanDiff	SEM	q Value	Prob	Alpha	Sig	LCL	UCL
30 nM SIK Control	3.20083	2.37555	1.90552	0.38949	0.05	0	-2.89326	9.29493
300 nM SIK Control	7.40417	2.37555	4.40785	0.01639	0.05	1	1.31007	13.49826
300 nM SIK 30 nM SIK	4.20333	2.53957	2.34072	0.25072	0.05	0	-2.31153	10.7182

Sig equals 1 indicates that the difference of the means is significant at the 0.05 level.
Sig equals 0 indicates that the difference of the means is not significant at the 0.05 level.

Powers

	Alpha	Sample Size	Power
Actual Power	0.05	20	0.72381
Hypothesis Powers	0.05	50	0.99321
	0.05	100	1
	0.05	200	1

Box Charts

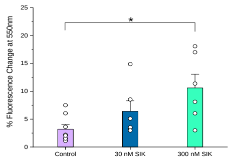

Supplement: Figure 2—source data 2. [file elife-65822-fig2-data2.pdf]
