# Supplementary material for: Voltage-clamp fluorometry analysis of structural rearrangements of ATP-gated channel P2X2 upon hyperpolarization
Source: eLife. 2021 May 19;10:e65822. doi: 10.7554/eLife.65822 (PMC8184218; doi:10.7554/eLife.65822)

ANOVAOneWay (23/12/2020 15:17:49)

Input Data

	Data	Range
control 2 days	[Book1]Sheet2!A"control 2 days"	[1*:12*]
control 3 days	[Book1]Sheet2!B"control 3 days"	[1*:8*]
cytoplasmic injection 2 days	[Book1]Sheet2!C"cytoplasmic injection 2 days"	[1*:8*]
cytoplasmic injection 3 days	[Book1]Sheet2!D"cytoplasmic injection 3 days"	[1*:5*]
nuclear injection 3 days	[Book1]Sheet2!E"nuclear injection 3 days"	[1*:6*]

Descriptive Statistics

	N Analysis	N Missing	Mean	Standard Deviation	SE of Mean
control 2 days	12	0	4.70833	1.86134	0.53732
control 3 days	8	0	3.1975	2.36339	0.83559
cytoplasmic injection 2 days	8	0	4.4725	2.054	0.7262
cytoplasmic injection 3 days	5	0	6.426	2.98513	1.33499
nuclear injection 3 days	6	0	10.60167	6.04239	2.4668

One Way ANOVA

Overall ANOVA

	DF	Sum of Squares	Mean Square	F Value	Prob>F
Model	4	218.80756	54.70189	5.72373	0.00124
Error	34	324.93907	9.55703		
Total	38	543.74663			

Null Hypothesis: The means of all levels are equal.
Alternative Hypothesis: The means of one or more levels are different.
At the 0.05 level, the population means are significantly different.

Fit Statistics

	R-Square	Coeff Var	Root MSE	Data Mean
	0.40241	0.56445	3.09144	5.47692

Means Comparisons

Tukey Test

	MeanDiff	SEM	q Value	Prob	Alpha	Sig	LCL	UCL
control 3 days control 2 days	-1.51083	1.41105	1.51423	0.82006	0.05	0	-5.574	2.55234
cytoplasmic injection 2 days control 2 days	-0.23583	1.41105	0.23636	0.99982	0.05	0	-4.299	3.82734
cytoplasmic injection 2 days control 3 days	1.275	1.54572	1.16652	0.92108	0.05	0	-3.17598	5.72598
cytoplasmic injection 3 days control 2 days	1.71767	1.64555	1.47619	0.83316	0.05	0	-3.02076	6.4561
cytoplasmic injection 3 days control 3 days	3.2285	1.76239	2.59067	0.37268	0.05	0	-1.8464	8.3034
cytoplasmic injection 3 days cytoplasmic injection 2 days	1.9535	1.76239	1.56756	0.80095	0.05	0	-3.1214	7.0284
nuclear injection 3 days control 2 days	5.89333	1.54572	5.39193	0.0047	0.05	1	1.44235	10.34431
nuclear injection 3 days control 3 days	7.40417	1.66957	6.27171	8.22124E-4	0.05	1	2.59656	12.21178
nuclear injection 3 days cytoplasmic injection 2 days	6.12917	1.66957	5.19172	0.00688	0.05	1	1.32156	10.93678
nuclear injection 3 days cytoplasmic injection 3 days	4.17567	1.87196	3.15459	0.1931	0.05	0	-1.21474	9.56607

Sig equals 1 indicates that the difference of the means is significant at the 0.05 level.
Sig equals 0 indicates that the difference of the means is not significant at the 0.05 level.

Powers

	Alpha	Sample Size	Power
Actual Power	0.05	39	0.96309
Hypothesis Powers	0.05	50	0.99252
	0.05	100	1
	0.05	200	1

Box Charts

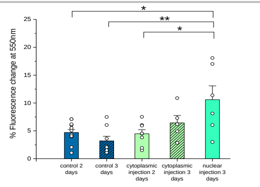

Supplement: Figure 2—source data 3. [file elife-65822-fig2-data3.pdf]
