# Supplementary material for: Voltage-clamp fluorometry analysis of structural rearrangements of ATP-gated channel P2X2 upon hyperpolarization
Source: eLife. 2021 May 19;10:e65822. doi: 10.7554/eLife.65822 (PMC8184218; doi:10.7554/eLife.65822)

Two sample t Test (23/12/2020 18:46:36)

Input Data

	Data	Range
1st Data Range	[Book1]Sheet1!A"Control"	[1*:7*]
2nd Data Range	[Book1]Sheet1!B"SIK inhibitor (300nM)"	[1*:12*]

Descriptive Statistics

		N	Mean	SD	SEM	Median
"Control"		7	0.77717	0.73843	0.2791	1.17764
"SIK inhibitor (300nM)"		12	0.83292	0.78954	0.22792	0.5043
	Difference		-0.05576		0.36711	
	Overall	19	0.81238	0.75065	0.17221	0.5098

Standard Error of Mean (SEM) of difference is computed under the condition that equal variance is assumed.

t-Test Statistics

	t Statistic	DF	Prob>|t|
Equal Variance Assumed	-0.15188	17	0.88107
Equal Variance NOT Assumed (Welch Correction)	-0.15473	13.41631	0.87934

Null Hypothesis: mean1-mean2 = 0
Alternative Hypothesis: mean1-mean2 <> 0
At 0.05 level, when equal variance is assumed, Mean1 - Mean2 is NOT significantly different from 0
At 0.05 level, when equal variance is NOT assumed, Mean1 - Mean2 is NOT significantly different from 0

Confidence Intervals for Mean

	Conf. Levels in %	Lower Limits	Upper Limits
	90	-0.69438	0.58286
	95	-0.83028	0.71877
	99	-1.11972	1.0082

Powers

	Alpha	Sample Size	Power
Actual Power	0.05	19	0.05236
Hypo. Power	0.05	50	0.05664
	0.05	100	0.0638
	0.05	200	0.07808

Plots

Box Plot

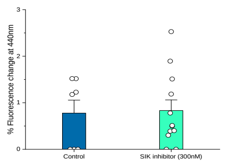

Supplement: Figure 2—source data 4. [file elife-65822-fig2-data4.pdf]
