# Supplementary material for: Voltage-clamp fluorometry analysis of structural rearrangements of ATP-gated channel P2X2 upon hyperpolarization
Source: eLife. 2021 May 19;10:e65822. doi: 10.7554/eLife.65822 (PMC8184218; doi:10.7554/eLife.65822)
Supplement: Figure 4—source data 2. [file elife-65822-fig4-data2.pdf]

Paired Sample t Test (16/04/2019 19:10:57)

Input Data

	Data	Range
1st Data Range	[Book1]"0+10uM ATP"!A"0 ATP"	[1*:4*]
2nd Data Range	[Book1]"0+10uM ATP"!B"10uM A TP"	[1*:4*]

Descriptive Statistics

		N	Mean	SD	SEM	Median
"0 ATP"		4	-1.92026	0.87334	0.43667	-1.77044
"10uM ATP"		4	-0.73892	0.30401	0.152	-0.77447
	Difference	4	-1.18134	0.59668	0.29834	-0.99597
	Overall	8	-1.32959	0.87477	0.30928	-1.04703

Test Statistics

	t Statistic	DF	Prob>|t|
	-3.95969	3	0.02876

Null Hypothesis: mean1-mean2 = 0
Alternative Hypothesis: mean1-mean2 <> 0
At the 0.05 level, the difference of the population means is significantly different from the test difference(0).

Confidence Intervals for Mean

	Conf. Levels in %	Lower Limits	Upper Limits
	90	-1.88345	-0.47923
	95	-2.13079	-0.23188
	99	-2.92392	0.56124

Powers

	Alpha	Sample Size	Power
Actual Power	0.05	4	0.74738
Hypo. Power	0.05	50	1
	0.05	100	1
	0.05	200	1

Plots

	Histogram	Box Plot
	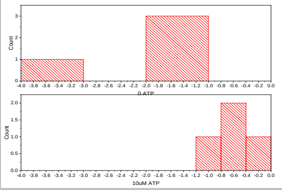	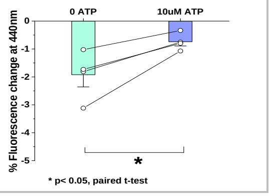
