# Supplementary material for: Voltage-clamp fluorometry analysis of structural rearrangements of ATP-gated channel P2X2 upon hyperpolarization
Source: eLife. 2021 May 19;10:e65822. doi: 10.7554/eLife.65822 (PMC8184218; doi:10.7554/eLife.65822)
Supplement: Figure 4—source data 3. [file elife-65822-fig4-data3.pdf]

Paired Sample t Test (17/04/2019 11:18:31)

Input Data

	Data	Range
1st Data Range	[Book1]"0+30uM ATP"!A"0 ATP"	[1*:8*]
2nd Data Range	[Book1]"0+30uM ATP"!B"30uM ATP"	[1*:8*]

Descriptive Statistics

		N	Mean	SD	SEM	Median
"0 ATP"		8	-2.65928	0.92277	0.32625	-2.68857
"30uM ATP"		8	-1.68962	0.60991	0.21564	-1.63125
	Difference	8	-0.96966	0.44321	0.1567	-0.84499
	Overall	16	-2.17445	0.90648	0.22662	-2.13188

Test Statistics

	t Statistic	DF	Prob>|t|
	-6.18808	7	4.50457E-4

Null Hypothesis: mean1-mean2 = 0

Alternative Hypothesis: mean1-mean2 <> 0

At the 0.05 level, the difference of the population means is significantly different from the test difference(0).

Confidence Intervals for Mean

	Conf. Levels in %	Lower Limits	Upper Limits
	90	-1.26654	-0.67278
	95	-1.34019	-0.59913
	99	-1.51803	-0.4213

Powers

	Alpha	Sample Size	Power
Actual Power	0.05	8	0.99943
Hypo. Power	0.05	50	1
	0.05	100	1
	0.05	200	1

Plots

	Histogram	Box Plot
	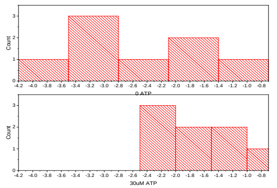	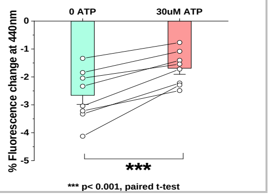
