# Supplementary material for: Voltage-clamp fluorometry analysis of structural rearrangements of ATP-gated channel P2X2 upon hyperpolarization
Source: eLife. 2021 May 19;10:e65822. doi: 10.7554/eLife.65822 (PMC8184218; doi:10.7554/eLife.65822)

Paired Sample t Test (16/04/2019 15:42:26)

Input Data

	Data	Range
1st Data Range	[Book1]"0+100uM ATP"!A"0 ATP"	[1*:8*]
2nd Data Range	[Book1]"0+100uM ATP"!B"100uM ATP"	[1*:8*]

Descriptive Statistics

		N	Mean	SD	SEM	Median
"0 ATP"		8	-2.42686	0.97287	0.34396	-2.18359
"100uM ATP"		8	-0.90564	0.42716	0.15102	-0.7687
	Difference	8	-1.52122	0.70733	0.25008	-1.43932
	Overall	16	-1.66625	1.06955	0.26739	-1.43058

Test Statistics

	t Statistic	DF	Prob>|t|
	-6.08297	7	4.99402E-4

Null Hypothesis: mean1-mean2 = 0
Alternative Hypothesis: mean1-mean2 <> 0
At the 0.05 level, the difference of the population means is significantly different from the test difference(0).

Confidence Intervals for Mean

	Conf. Levels in %	Lower Limits	Upper Limits
	90	-1.99502	-1.04743
	95	-2.11257	-0.92988
	99	-2.39637	-0.64608

Powers

	Alpha	Sample Size	Power
Actual Power	0.05	8	0.99923
Hypo. Power	0.05	50	1
	0.05	100	1
	0.05	200	1

Plots

	Histogram	Box Plot

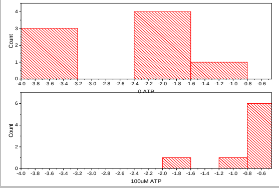

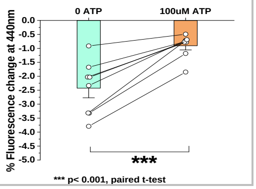

Supplement: Figure 4—source data 4. [file elife-65822-fig4-data4.pdf]
