# Supplementary material for: Voltage-clamp fluorometry analysis of structural rearrangements of ATP-gated channel P2X2 upon hyperpolarization
Source: eLife. 2021 May 19;10:e65822. doi: 10.7554/eLife.65822 (PMC8184218; doi:10.7554/eLife.65822)
Supplement: Figure 4—figure supplement 1—source data 2. [file elife-65822-fig4-figsupp1-data2.pdf]

Paired Sample t Test (13/04/2019 17:47:14)

Input Data

	Data	Range
1st Data Range	[Book1]"0+30uM ATP"!A"0 ATP"	[1*:5*]
2nd Data Range	[Book1]"0+30uM ATP"!B"30uM ATP"	[1*:5*]

Descriptive Statistics

		N	Mean	SD	SEM	Median
"0 ATP"		5	-2.78579	1.68882	0.75526	-1.99926
"30uM ATP"		5	-1.8153	0.95568	0.42739	-1.58223
	Difference	5	-0.97049	0.8933	0.3995	-0.64928
	Overall	10	-2.30055	1.3911	0.4399	-1.86732

Test Statistics

	t Statistic	DF	Prob>|t|
	-2.42928	4	0.07204

Null Hypothesis: mean1-mean2 = 0
Alternative Hypothesis: mean1-mean2 <> 0
At the 0.05 level, the difference of the population means is NOT significantly different from the test difference(0).

Confidence Intervals for Mean

	Conf. Levels in %	Lower Limits	Upper Limits
	90	-1.82216	-0.11883
	95	-2.07967	0.13869
	99	-2.80981	0.86883

Powers

	Alpha	Sample Size	Power
Actual Power	0.05	5	0.4568
Hypo. Power	0.05	50	1
	0.05	100	1
	0.05	200	1

Plots

	Histogram	Box Plot
	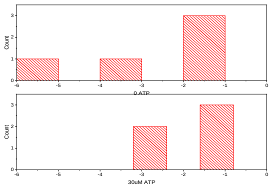	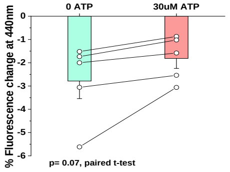
