# Supplementary material for: Voltage-clamp fluorometry analysis of structural rearrangements of ATP-gated channel P2X2 upon hyperpolarization
Source: eLife. 2021 May 19;10:e65822. doi: 10.7554/eLife.65822 (PMC8184218; doi:10.7554/eLife.65822)

Paired Sample t Test (13/04/2019 17:47:28)

Input Data

	Data	Range
1st Data Range	[Book1]"0+100uM ATP"!A"0 ATP"	[1*:5*]
2nd Data Range	[Book1]"0+100uM ATP"!B"100uM ATP"	[1*:5*]

Descriptive Statistics

		N	Mean	SD	SEM	Median
"0 ATP"		5	-2.6538	0.97417	0.43566	-2.59239
"100uM ATP"		5	-0.997	0.43184	0.19312	-0.97998
	Difference	5	-1.6568	0.6585	0.29449	-1.59295
	Overall	10	-1.8254	1.12568	0.35597	-1.61207

Test Statistics

	t Statistic	DF	Prob>|t|
	-5.62604	4	0.00491

Null Hypothesis: mean1-mean2 = 0
Alternative Hypothesis: mean1-mean2 <> 0
At the 0.05 level, the difference of the population means is significantly different from the test difference(0).

Confidence Intervals for Mean

	Conf. Levels in %	Lower Limits	Upper Limits
	90	-2.28461	-1.029
	95	-2.47443	-0.83917
	99	-3.01266	-0.30095

Powers

	Alpha	Sample Size	Power
Actual Power	0.05	5	0.98278
Hypo. Power	0.05	50	1
	0.05	100	1
	0.05	200	1

Plots

	Histogram	Box Plot
	See below	See below

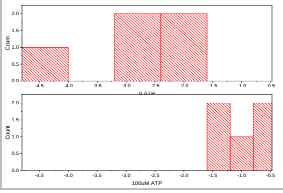

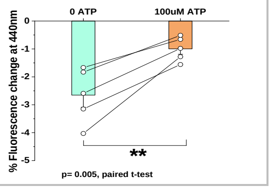

Supplement: Figure 4—figure supplement 1—source data 3. [file elife-65822-fig4-figsupp1-data3.pdf]
