# Supplementary material for: Voltage-clamp fluorometry analysis of structural rearrangements of ATP-gated channel P2X2 upon hyperpolarization
Source: eLife. 2021 May 19;10:e65822. doi: 10.7554/eLife.65822 (PMC8184218; doi:10.7554/eLife.65822)
Supplement: Figure 5—figure supplement 2—source data 2. [file elife-65822-fig5-figsupp2-data2.pdf]

Table Analyzed	time constant 30 vs 300			
Column B	300 µM ATP			
vs.	vs.			
Column A	30 µM ATP			
Unpaired t test				
P value	<0.0001			
P value summary	****			
Significantly different (P < 0.05)?	Yes			
One- or two-tailed P value?	Two-tailed			
t	df		t=7.154	df=8
How big is the difference?				
Mean of column A		70.41		
Mean of column B		26.9		
Difference between means (B - A) ± SEM	-43.51 ± 6.081			
95% confidence interval	-57.53 to -29.48			
R squared (eta squared)		0.8648		
F test to compare variances				
F	DFn		Dfd	2.923
P value		0.5534		
P value summary	ns			
Significantly different (P < 0.05)?	No			
Data analyzed				
Sample size	column A		3	
Sample size	column B		7	

6 2
