# Supplementary material for: Voltage-clamp fluorometry analysis of structural rearrangements of ATP-gated channel P2X2 upon hyperpolarization
Source: eLife. 2021 May 19;10:e65822. doi: 10.7554/eLife.65822 (PMC8184218; doi:10.7554/eLife.65822)
Supplement: Figure 6—figure supplement 1—source data 2. [file elife-65822-fig6-figsupp1-data2.pdf]

Table Analyzed	0 ATP vs 300 µM ATP batch 1 VCF A	
Column B	300 µM ATP	
vs.	vs.	
Column A	0 ATP	
Paired t test		
P value		0.0327
P value summary	*	
Significantly different ($P < 0.05$)?	Yes	
One- or two-tailed P value?	Two-tailed	
t	df	t=3.205
Number of pairs		5
How big is the difference?		
Mean of differences (B - A)		-0.1621
SD of differences		0.1131
SEM of differences		0.05058
95% confidence interval	-0.3026 to -0.02170	
R squared (partial eta squared)		0.7198
How effective was the pairing?		
Correlation coefficient (r)		0.9992
P value (one tailed)	<0.0001	
P value summary	****	
Was the pairing significantly effective?	Yes	

df=4
