# Supplementary material for: Voltage-clamp fluorometry analysis of structural rearrangements of ATP-gated channel P2X2 upon hyperpolarization
Source: eLife. 2021 May 19;10:e65822. doi: 10.7554/eLife.65822 (PMC8184218; doi:10.7554/eLife.65822)
Supplement: Figure 7—source data 2. [file elife-65822-fig7-data2.pdf]

Paired Sample t Test (13/04/2019 18:22:26)

Input Data

	Data	Range
1st Data Range	[Book1]WT!A"0 ATP"	[1*:5*]
2nd Data Range	[Book1]WT!B"30µM ATP"	[1*:5*]

Descriptive Statistics

		N	Mean	SD	SEM	Median
"0 ATP"		5	-0.552	0.19917	0.08907	-0.52
"30µM ATP"		5	-10.484	1.19843	0.53595	-10.4
	Difference	5	9.932	1.31669	0.58884	9.9
	Overall	10	-5.518	5.29691	1.67503	-4.995

Test Statistics

	t Statistic	DF	Prob>|t|
	16.86703	4	7.2425E-5

Null Hypothesis: mean1-mean2 = 0

Alternative Hypothesis: mean1-mean2 <> 0

At the 0.05 level, the difference of the population means is significantly different from the test difference(0).

Confidence Intervals for Mean

	Conf. Levels in %	Lower Limits	Upper Limits
	90	8.67668	11.18732
	95	8.29711	11.56689
	99	7.22092	12.64308

Powers

	Alpha	Sample Size	Power
Actual Power	0.05	5	1
Hypo. Power	0.05	50	1
	0.05	100	1
	0.05	200	1

Plots

	Histogram	Box Plot
	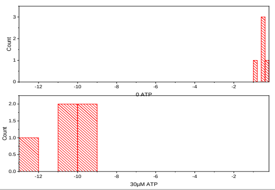	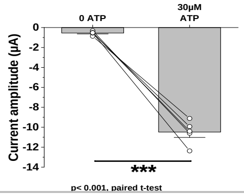

Paired Sample t Test (13/04/2019 18:33:41)

Input Data

	Data	Range
1st Data Range	[Book1]F44E!A"0 ATP"	[1*:4*]
2nd Data Range	[Book1]F44E!B"30µM ATP"	[1*:4*]

Descriptive Statistics

		N	Mean	SD	SEM	Median
"0 ATP"		4	-0.63	0.38557	0.19279	-0.7
"30µM ATP"		4	-8.15	1.48151	0.74075	-7.935
	Difference	4	7.52	1.1465	0.57325	7.375
	Overall	8	-4.39	4.14266	1.46465	-3.92

Test Statistics

	t Statistic	DF	Prob>|t|
	13.11816	3	9.56842E-4

Null Hypothesis: mean1-mean2 = 0
Alternative Hypothesis: mean1-mean2 <> 0
At the 0.05 level, the difference of the population means is significantly different from the test difference(0).

Confidence Intervals for Mean

	Conf. Levels in %	Lower Limits	Upper Limits
	90	6.17092	8.86908
	95	5.69566	9.34434
	99	4.17169	10.86831

Powers

	Alpha	Sample Size	Power
Actual Power	0.05	4	1
Hypo. Power	0.05	50	1
	0.05	100	1
	0.05	200	1

Plots

	Histogram	Box Plot
	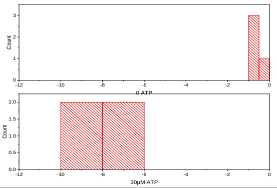	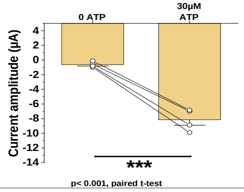

Paired Sample t Test (13/04/2019 18:18:17)

Input Data

	Data	Range
1st Data Range	[Book1]F44E/A337R!A"0 ATP"	[1*:4*]
2nd Data Range	[Book1]F44E/A337R!B"30µM ATP "	[1*:4*]

Descriptive Statistics

		N	Mean	SD	SEM	Median
"0 ATP"		4	-3.935	2.30623	1.15312	-4.04
"30µM ATP"		4	-4.0275	2.35333	1.17666	-4.17
	Difference	4	0.0925	0.09251	0.04626	0.055
	Overall	8	-3.98125	2.15763	0.76284	-4.055

Test Statistics

	t Statistic	DF	Prob>|t|
	1.99976	3	0.13936

Null Hypothesis: mean1-mean2 = 0
Alternative Hypothesis: mean1-mean2 <> 0
At the 0.05 level, the difference of the population means is NOT significantly different from the test difference(0).

Confidence Intervals for Mean

	Conf. Levels in %	Lower Limits	Upper Limits
	90	-0.01636	0.20136
	95	-0.05471	0.23971
	99	-0.17767	0.36267

Powers

	Alpha	Sample Size	Power
Actual Power	0.05	4	0.2887
Hypo. Power	0.05	50	1
	0.05	100	1
	0.05	200	1

Plots

	Histogram	Box Plot
	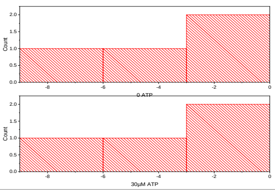	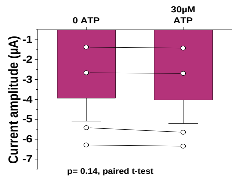
