# Supplementary material for: Voltage-clamp fluorometry analysis of structural rearrangements of ATP-gated channel P2X2 upon hyperpolarization
Source: eLife. 2021 May 19;10:e65822. doi: 10.7554/eLife.65822 (PMC8184218; doi:10.7554/eLife.65822)
Supplement: Supplementary file 1. — Mutations were introduced one at a time into 96 positions within the extracellular domain (ECD) near the ATP-binding site and extracellular linker, transmembrane domains (TMs), intracellular N-terminal, and intracellular C-terminal. ATP application ranging from 10 µM, 30 µM, or 100 µM unless otherwise stated. (+) indicates there was either ATP-evoked fluorescence (F) signal change, voltage-evoked F change, ATP-evoked current (I) change, or voltage-evoked I change. (-) indicates negative results. (**) indicates mutants which have a very low expression level, so that the reliable VCF analysis could not be undertaken. (***) indicates fast current decay. (--) indicates that the subsequent recording could not be performed, as a result of fast current decay. (n.d.) indicates not determined. [file elife-65822-supp1.docx]

**Supplementary Table 1**

| **No.** | **Domain** | **TAG mutation position** | **Fluorescence (F) change** | | **Current (I) change** | |
| --- | --- | --- | --- | --- | --- | --- |
|  |  |  | **ATP-**  **evoked**  **F change** | **Voltage-**  **evoked**  **F change** | **ATP-**  **evoked**  **I change** | **Voltage-evoked**  **I change** |
| 1 | Extracellular Domain (ECD), around ATP-binding site | D209 | + | - | + | + |
| 2 |  | A283 | + | - | + | + |
| 3 |  | S284 | - | - | - | - |
| 4 |  | S285 | n.d. | - | + | + |
| 5 |  | G286 | n.d. | - | + | + |
| 6 | ECD,  in extracellular linker | K53 | + | - | + | + |
| 7 |  | S54 | + | - | + | + |
| 8 |  | Y55 (**) | n.d. | - | + | + |
| 9 |  | S58 | + | - | + | + |
| 10 |  | E63 | - | - | + | + |
| 11 |  | S65 | - | - | - | - |
| 12 |  | I67 | - | - | + | + |
| 13 |  | E91 | n.d. | - | + | + |
| 14 |  | T105 | n.d. | - | + | + |
| 15 |  | E167 | n.d. | - | + | + |
| 16 |  | R304 | n.d. | - | + | + |
| 17 |  | A309 | n.d. | - | + | + |
| 18 |  | R313 | - | - | + | + |
| 19 |  | I314 | n.d. | - | + | + |
| 20 |  | D315 | + | - | + | + |
| 21 |  | V316 | - | - | - | - |
| 22 |  | I317 | - | - | + | + |
| 23 |  | V318 | - | - | - | - |
| 24 |  | H319 | - | - | + | + |
| 25 |  | A322 | n.d. | - | + | + |
| 26 |  | K324 | - | - | + | + |
| 27 |  | F325 | + | - | + | + |
| 28 | Transmembrane 2 | S326 | + | - | + | + |
| 29 |  | L327 | - | - | + | + |
| 30 |  | I328 | - | - | + | - |
| 31 |  | P329 | - | - | + | + |
| 32 |  | T330 | - | - | + | + |
| 33 |  | I331 | + | - | + | + |
| 34 |  | I332 | + | - | + | + |
| 35 |  | N333 | - | - | + | + |
| 36 |  | L334 | - | - | + | + |
| 37 |  | A335 | + | - | + | + |
| 38 |  | T336 | - | - | - | - |
| 39 |  | A337 | + | + | + | + |
| 40 |  | L338 | - | - | + | + |
| 41 |  | T339 | - | - | + | - |
| 42 |  | S340 (**) | - | - | - | - |
| 43 |  | I341 | + | + | + | + |
| 44 |  | G342 (**) | - | - | - | - |
| 45 |  | V343 | + | - | + | + |
| 46 |  | G344 | - | - | +* (1mM) | +* (1mM) |
| 47 |  | S345 | - | - | + | + |
| 48 |  | F346 | + | - | + | + |
| 49 |  | L347 | - | - | + | + |
| **No.** | **Domain** | **TAG mutation position** | **Fluorescence (F) change** | | **Current (I) change** | |
|  |  |  | **ATP-**  **evoked**  **F change** | **Voltage-**  **evoked**  **F change** | **ATP-**  **evoked**  **I change** | **Voltage-evoked**  **I change** |
| 50 | Transmembrane 2 | C348 | - | - | - | - |
| 51 |  | D349 | - | - | - | - |
| 52 | Transmembrane 1 | R34 | n.d. | - | + | + |
| 53 |  | M35 | + | - | + | + |
| 54 |  | V36 | + | - | + | + |
| 55 |  | Q37 | + | - | + | + |
| 56 |  | L38 | n.d. | - | + | + |
| 57 |  | L39 | n.d. | - | + | + |
| 58 |  | I40 | n.d. | - | + | + |
| 59 |  | L41 | n.d. | - | + | + |
| 60 |  | L42 | n.d. | - | + | + |
| 61 |  | Y43 | + | - | + | - |
| 62 |  | F44 | - | - | - | - |
| 63 |  | V45 | + | - | + | + |
| 64 |  | W46 | + | - | + | + |
| 65 |  | Y47 | + | - | + | - |
| 66 |  | V48 | - | - | - | - |
| 67 |  | F49 | + | - | + | + |
| 68 |  | I50 | + | - | + | + |
| 69 |  | V51 | + | - | + | + |
| 70 |  | Q52 | - | - | + | + |
| 71 | Intracellular  C terminal | W350 | - | - | - | - |
| 72 |  | I351 | - | - | + (3mM) | + (3mM) |
| 73 |  | F355 | - | - | + | + |
| 74 |  | M356 | - | -- | + (***) | -- |
| 75 |  | N357 | - | - | - | - |
| 76 |  | K358 | - | -- | + (***) | -- |
| 77 |  | N359 | - | - | + | + |
| 78 |  | L361 | - | - | + | + |
| 79 |  | Y362 | - | -- | + (***) | -- |
| 80 |  | S363 | - | -- | + (***) | -- |
| 81 |  | H364 | - | - | + | + |
| 82 |  | F367 | - | - | - | - |
| 83 |  | D368 | - | - | - | - |
| 84 |  | V370 | - | -- | + (***) | -- |
| 85 |  | R371 | + | - | + | + |
| 86 |  | T372 | - | - | + | + |
| 87 |  | P373 | + | - | + | + |
| 88 |  | K374 | + | - | + | + |
| 89 | Intracellular  N terminal | Y16 | - | - | - | - |
| 90 |  | E17 | - | - | - | - |
| 91 |  | T18 | - | - | - | - |
| 92 |  | P19 | - | - | - | - |
| 93 |  | K20 | - | - | - | - |
| 94 |  | V24 | - | -- | + (***) | -- |
| 95 |  | N26 | - | -- | + (***) | + |
| 96 |  | V32 | - | - | + | + |
