# Supplementary material for: Voltage-clamp fluorometry analysis of structural rearrangements of ATP-gated channel P2X2 upon hyperpolarization
Source: eLife. 2021 May 19;10:e65822. doi: 10.7554/eLife.65822 (PMC8184218; doi:10.7554/eLife.65822)
Supplement: Transparent reporting form [file elife-65822-transrepform.docx]

***eLife’s* transparent reporting form**

We encourage authors to provide detailed information *within their submission* to facilitate the interpretation and replication of experiments. Authors can upload supporting documentation to indicate the use of appropriate reporting guidelines for health-related research (see [EQUATOR Network](http://www.equator-network.org/%20)), life science research (see the [BioSharing Information Resource](https://biosharing.org/)), or the [ARRIVE guidelines](http://www.plosbiology.org/article/info:doi/10.1371/journal.pbio.1000412) for reporting work involving animal research. Where applicable, authors should refer to any relevant reporting standards documents in this form.

If you have any questions, please consult our Journal Policies and/or contact us: [editorial@elifesciences.org](mailto:editorial@elifesciences.org).

**Sample-size estimation**

- You should state whether an appropriate sample size was computed when the study was being designed
- You should state the statistical method of sample size computation and any required assumptions
- If no explicit power analysis was used, you should describe how you decided what sample (replicate) size (number) to use

Please outline where this information can be found within the submission (e.g., sections or figure legends), or explain why this information doesn’t apply to your submission:

Statistical method was not used for sample size calculation

Number of successfully recorded data used for analyses: 3-6 for TEVC; 3-12 for VCF

Batch used for recording: 2-3 for TEVC; 3-10 for VCF

Oocytes used for recording from each batch: 5-10 for TEVC; 5-15 for VCF

Note:

(1) In TEVC experiments, data of unsuccessful recordings (too low expression level, leaky, too high expression level, insufficient voltage clamp) were not used for the analyses.

(2) In VCF experiments, the successful detection of fluorescence (F) change significantly varied between batches, probably due to e.g., the level of background fluorescence signal. Data from batches in which F changes were detected were used for the analyses.

**Replicates**

- You should report how often each experiment was performed
- You should include a definition of biological versus technical replication
- The data obtained should be provided and sufficient information should be provided to indicate the number of independent biological and/or technical replicates
- If you encountered any outliers, you should describe how these were handled
- Criteria for exclusion/inclusion of data should be clearly stated
- High-throughput sequence data should be uploaded before submission, with a private link for reviewers provided (these are available from both GEO and ArrayExpress)

Please outline where this information can be found within the submission (e.g., sections or figure legends), or explain why this information doesn’t apply to your submission:

We define biological replication as the variation between different batches of the oocytes, and technical replication as the variation between different cells in the same batch of the oocytes. We performed successful VCF experiments in 2-8 batches meanwhile TEVC recordings in 2-3 batches.

Outliers are defined by:

(1) In TEVC experiments, data of unsuccessful recordings (too low expression level, leaky, too high expression level, insufficient voltage clamp) were not used for the analyses.

(2) In VCF experiments, the successful detection of fluorescence (F) change significantly varied between batches, probably due to e.g., the level of background fluorescence signal. Data from batches in which F changes were detected were used for the analyses.

**Statistical reporting**

- Statistical analysis methods should be described and justified
- Raw data should be presented in figures whenever informative to do so (typically when N per group is less than 10)
- For each experiment, you should identify the statistical tests used, exact values of N, definitions of center, methods of multiple test correction, and dispersion and precision measures (e.g., mean, median, SD, SEM, confidence intervals; and, for the major substantive results, a measure of effect size (e.g., Pearson's r, Cohen's d)
- Report exact p-values wherever possible alongside the summary statistics and 95% confidence intervals. These should be reported for all key questions and not only when the p-value is less than 0.05.

Please outline where this information can be found within the submission (e.g., sections or figure legends), or explain why this information doesn’t apply to your submission:

Information about statistical analysis method can be found in the figure legends of: Figure 2, Figure 4, Figure 4—figure supplement 1, Figure 5—figure supplement 2, Figure 6—figure supplement 1, and Figure 7.

Further information about statistical analysis method can be found in materials method section: statistical analysis (performed by either OriginPro or GraphPad Prism 9)

Results of statistical analysis can be found in Figure 2-source data 2, Figure 2-source data 3, Figure 2-source data 4, Figure 4-source data 2, Figure 4-source data 3, Figure 4-source data 4, Figure 4—figure supplement 1-source data 2, Figure 4—figure supplement 1-source data 3, Figure 5—figure supplement 2-source data 2, Figure 6—figure supplement 1-source data 2, and Figure 7-source data 2.

(For large datasets, or papers with a very large number of statistical tests, you may upload a single table file with tests, Ns, etc., with reference to sections in the manuscript.)

**Group allocation**

- Indicate how samples were allocated into experimental groups (in the case of clinical studies, please specify allocation to treatment method); if randomization was used, please also state if restricted randomization was applied
- Indicate if masking was used during group allocation, data collection and/or data analysis

Please outline where this information can be found within the submission (e.g., sections or figure legends), or explain why this information doesn’t apply to your submission:

N/A

**Additional data files (“source data”)**

- We encourage you to upload relevant additional data files, such as numerical data that are represented as a graph in a figure, or as a summary table
- Where provided, these should be in the most useful format, and they can be uploaded as “Source data” files linked to a main figure or table
- Include model definition files including the full list of parameters used
- Include code used for data analysis (e.g., R, MatLab)
- Avoid stating that data files are “available upon request”

Please indicate the figures or tables for which source data files have been provided:

Source data file of each graph is attached (Figure 1, Figure 1—figure supplement 1, Figure 2, Figure 2—figure supplement 1, Figure 3, Figure 3—figure supplement 1, Figure 4, Figure 4—figure supplement 1, Figure 5, Figure 5—figure supplement 1, Figure 5—figure supplement 2, Figure 6, Figure 6—figure supplement 1 and Figure 7)
